# Anesthetic Management of an Adult Patient With Down Syndrome for Thoracic Surgery

**DOI:** 10.7759/cureus.17130

**Published:** 2021-08-12

**Authors:** Massimiliano Pelli, Chiara Loffredo, Cecilia Menna, Silvia Fiorelli, Domenico Massullo

**Affiliations:** 1 Division of Anesthesia and Intensive Care Medicine, Department of Clinical and Surgical Translational Medicine, Sant' Andrea Hospital, Sapienza University of Rome, Rome, ITA; 2 Division of Thoracic Surgery, Department of Clinical and Surgical Translational Medicine, Sant' Andrea Hospital, Sapienza University of Rome, Rome, ITA

**Keywords:** anesthesia, thoracic surgery, one-lung isolation, down syndrome, trisomy 21

## Abstract

The anesthetic management of adult patient with Down syndrome (DS) can be challenging due to poor patient cooperation, age-related comorbidities, and a possible difficult airway. Thoracic anesthesia requires an advanced airway management; thus, treatment of DS patients can be particularly demanding. An accurate preoperative assessment is paramount in order to plan a well-designed perioperative strategy in advance. This report describes the anesthetic management of an adult patient affected by DS who underwent pleural decortication for pleural empyema.

## Introduction

Down syndrome (DS) is the most common genetic disease involving chromosome 21, and it is characterized by multiple comorbidities, first of all cognitive disability [1]. Life expectancy of these patients has improved over the years due to enhanced medical care, thus increasing the need for surgical interventions [2]. Cardiovascular abnormalities, respiratory diseases, obstructive sleep apnea syndrome, atlantoaxial instability, overweight, cranio-facial abnormalities, and mental and behavioral disorders make these patients a challenge for the anesthetic management [3].

The major causes of death in adult DS patients are cardiovascular and respiratory diseases, the latter accounting for 20-40% of cases. In this scenario, thoracic surgery may assume a paramount role, requiring particular expertise like one-lung ventilation (OLV), and double-lumen tube (DLT) or bronchial blocker (BB) positioning. Given the lack of literature about this topic, the anesthetic management in a 55-year-old patient affected by DS who underwent pleural decortication for pleural empyema is described.

## Case presentation

**Figure 1:**
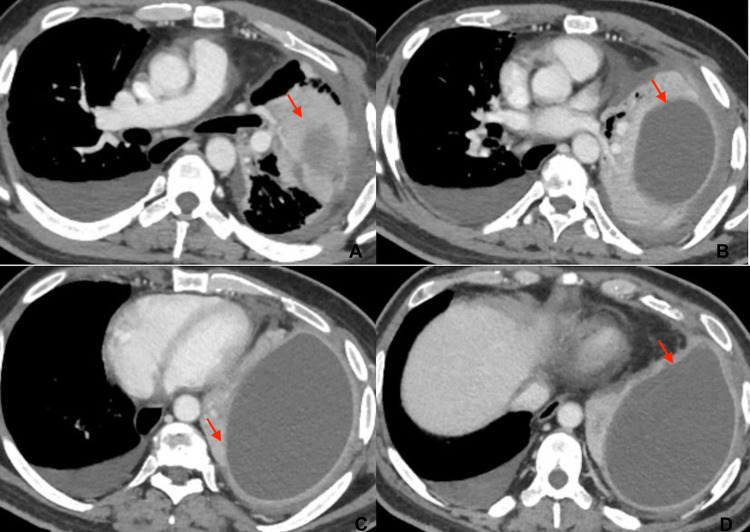
A-D. Axial sections of chest CT scan showing a left chronic thick-walled Stage III empyema.

The patient was admitted to the Thoracic Surgery Unit because of left pleural empyema and scheduled for pleural decortication with pleural toilet. He was 55 years old, 175 cm tall, and weighed 70 kg. No comorbidities or previous surgical interventions were reported. Written informed consent was obtained from the patient before surgery. The patient was agitated, anxious, poorly cooperative, and mentally disabled. He poorly understood verbal communication and showed difficulty in speech. He attended the anesthetic presurgical evaluation with his brother (guardian), although he was not legally incompetent. The patient underwent complete blood count, comprehensive metabolic panel, and coagulation panel, without any reported pathological values. Given the high prevalence of cardiac abnormalities in DS patients, a cardiology consultancy was performed. No cardiac signs or symptoms were detected, electrocardiogram was normal, and transthoracic echocardiography showed a normal ejection fraction with no valvular defects. Preoperative chest CT scan showed a left chronic thick-walled Stage III empyema (organizing stage, Figure 1) requiring toilet of the chest cavity and pleural decortication through thoracotomy. Lung atelectasis of the entire left lower lobe and the most of the left upper lobe were present. A minimal amount of contralateral pleural effusion was also found.

The patient was classified according to American Society of Anesthesiology classification as class 3, considering his mild functional limitations. A careful airway evaluation was performed, considering the patient at high risk for difficult mask ventilation (DMV) and difficult intubation (DI). Specifically, interincisors distance was 3 cm, with relative macroglossia, thyromental distance was 6 cm, microstomia was present, as well as poor neck mobility. Moreover, modified Mallampati score was grade IV (only hard palate was visible at mouth opening), upper lip bite test was at high risk (inability to reach a contact between mandibular teeth and the vermilion border), and the typical craniofacial abnormalities of DS patients were present. STOP-BANG score was 3.

The following anesthetic management was planned: induction of general anesthesia, positioning of a supraglottic airway device, preoperative bronchoscopy to exclude possible tracheal-bronchial abnormalities, and bronchoscopy-guided tracheal intubation with single-lumen tube (SLT) and placement of a BB.

**Figure 2:**
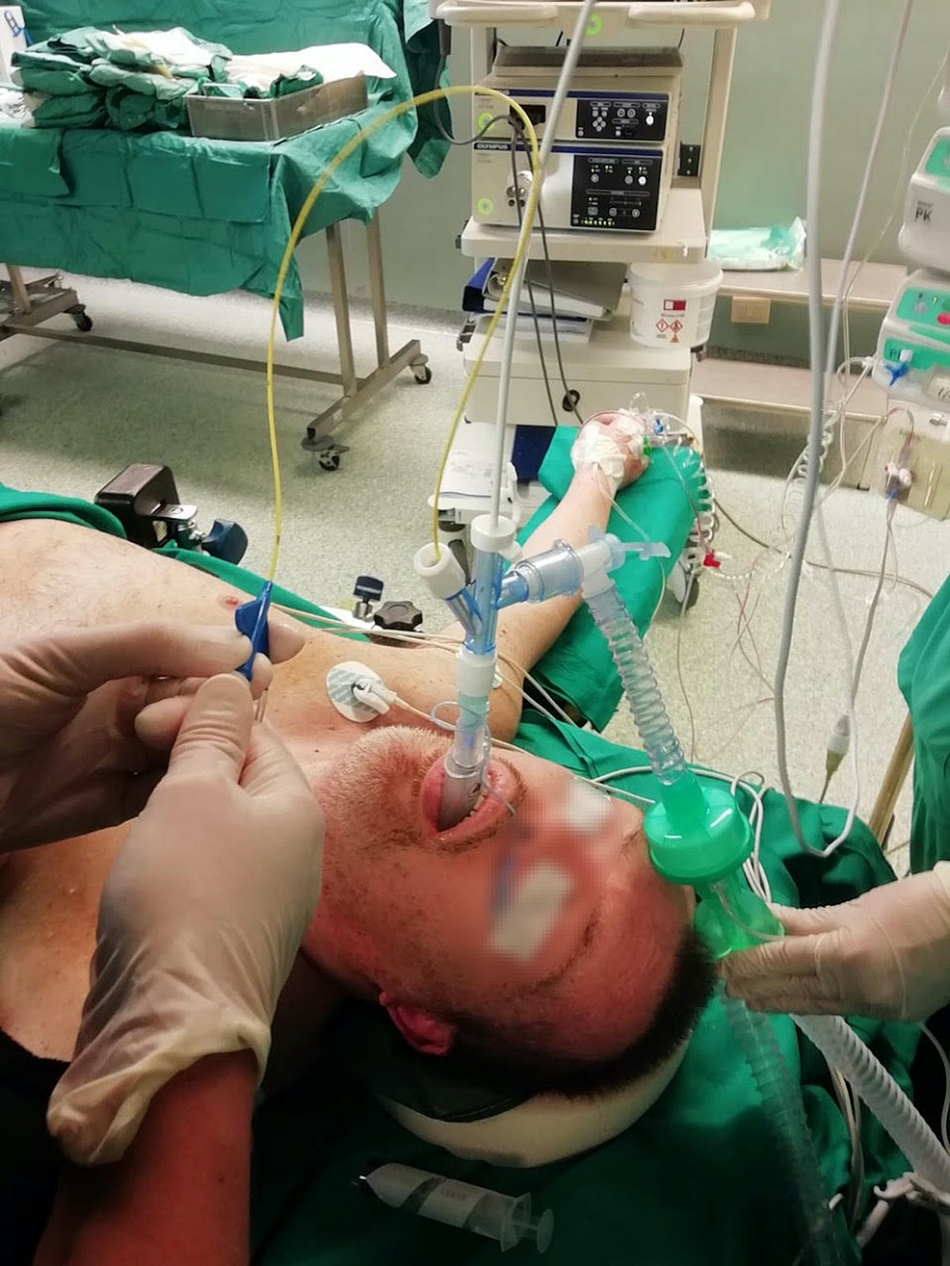
One-lung isolation obtained with a single-lumen tube passed through a laryngeal mask and a bronchial blocker positioning.

On the day of surgery, the patient appeared agitated and uncooperative (Richmond Agitation Sedation Scale [RASS] +3). Sixty minutes before surgery oral ketamine 1.4 mg/kg and midazolam 0.2 mg/kg were administered. On arrival in the operating room, the patient was lightly sedated (RASS −2). Venous access was not difficult and an 18-gauge IV catheter was secured. Right radial artery cannulation for invasive intraoperative blood pressure monitoring was also performed. A standard electrocardiogram monitoring, pulse oximeter, invasive blood pressure, and train of four (TOF) on the right ulnar nerve were placed. At induction propofol 100 mg, fentanyl 100 mcg, and rocuronium 50 mg were administered. DMV occurred, so an I-gel laryngeal mask airway (LMA, Intersurgical, UK) size 4 was placed in the neutral head position, obtaining a satisfactory ventilation. The preoperative bronchoscopy was performed by the surgeon during ventilatory assistance through LMA using a single-use flexible endoscope (Ambu® aScope 4, Ambu A/S, Ballerup, Denmark). Neither tracheal nor main bronchi lesions were observed. Successively, tracheal intubation with an SLT size 7 was performed through LMA under bronchoscopic vision. Lung isolation was ensured with Arndt 9.0 French endobronchial blocker (Cook Medical, Bloomington, USA) inserted into the left main bronchus with flexible bronchoscope assistance (Figure 2).

Dexamethasone 4 mg, ondasetron 8 mg, ketorolac 30 mg, and cefazoline 2 g were administered before the incision. Maintenance of general anesthesia was achieved with desflurane (1 minimum alveolar concentration) and continuous infusion of remifentanil, with a target-controlled infusion system in effect-site target mode with an Alaris® PK syringe pump. Mechanical ventilation was set in volume-controlled ventilation (VCV): tidal volume (Vt) 400 mL, respiratory rate 12 breaths per minute, positive end-expiratory pressure 7 cmH_2_O, inspiratory time 1.7 seconds, oxygen inspiratory fraction 0.65. Mechanical ventilation was set in order to have the lower driving pressure with the most satisfactory SpO_2_ (94-95%). Two cycling recruitment maneuvers were applied after the beginning and at the end of OLV. OLV duration was 100 minutes. A thoracoscopic approach was performed to explore the chest cavity. The presence of tough pleural adhesions with a nearly total pleural space obliteration required a conversion to an open thoracotomy. Adequate pleural decortication and surgical toilet of the chest cavity were performed, removing the empyematous sac. Operative time was 120 minutes. Two chest tube drainages were placed. The patient received intrapleural intercostal nerve block from the fourth to the eighth space performed by the surgeon under direct vision from inside the chest, with 20 mL of 0.75% ropivacaine, 4 mL for each space. Paracetamol 1 g and morphine 3 mg were administered 30 minutes before the end of the surgery. Finally, BB was removed, the TOF tests revealed 4/4 strong twitches, and complete neuromuscular reversal was achieved with 200 mg of sugammadex. LMA and SLT were removed. Total intraoperative fluid administered was 4.5 mL/kg/h, blood loss was about 100 mL, and urine output was 100 mL/h. After surgery, the patient remained in the recovery room for 30 minutes. He was spontaneously breathing ambient air with SpO_2_ 94%, vital signs were within normal range, and he appeared drowsy and calm (RASS −1). A 24-hour IV elastomeric pump with ketorolac 90 mg, tramadol 300 mg, and metoclopramide 20 mg at infusion rate or 2 mL/h was placed. Pain assessment was performed using COMFORT-Behavior (COMFORT-B) scale. The day after surgery he could walk with the assistance of his guardian and without pain. He was discharged from the hospital after six days without any surgical or medical complications.

## Discussion

Premedication with ketamine and midazolam enabled the application of planned anesthetic management in a difficult-airway adult DS patient. LMA placement and subsequent SLT and BB positioning allowed to secure the patient’s difficult airway and to perform OLV effectively. Previous reports about anesthetic management of patients affected by DS have focused mainly on pediatric patients, and several topics have been highlighted, such as difficult airways due to the presence of subglottic stenosis, macroglossia, tracheomalacia, joint immobility, and also demanding IV access placement [3] . In older patients with DS, an increased prevalence of comorbidities can rise additional perioperative issues.

One of the major concerns in mentally disabled patients is poor cooperativity and aggressive behavior that can make it challenging to implement appropriate anesthetic management. Combined oral use of ketamine and midazolam has already demonstrated to be effective in children and uncooperative patients [4], because of having amnesic, sedative, and anxiolytic properties. Moreover, the co-administration eliminates the psychotomimetic and cardiovascular effects of ketamine [5]; in fact, Funk et al. demonstrated that this combination had fewer side effects than the two drugs given alone [6]. Oral administration in combative patients is convenient, effective, and safe for both drugs. Hanamoto et al. showed that oral midazolam was more efficient than that provided through intramuscular route [7], while ketamine had good oral bioavailability and norketamine, its first-pass metabolite, was fully active with analgesic properties [8]. Even if lower doses of ketamine and midazolam than those reported in literature were used [4-8], easy preoperative management was allowed, without delaying patient’s extubation and postoperative recovery time, as already demonstrated by Trabold et al. in children [9]. The patient did not experience postoperative nausea and vomiting, thanks to intraoperative low opioid use and dexamethasone and ondansetron prophylaxis [10]. 

The patient was considered at high risk for DMV and DI, according to multiple airway features [11]. In a preplanned airway strategy, awake intubation was excluded because of poor patient collaboration [12]. LMA positioning, after DMV occurrence at induction [11], provided adequate ventilation, and tracheal intubation could be performed under bronchoscopic guide, thus securing the difficult airways and excluding small subglottic area and tracheal stenosis, a quite frequent event in DS patients [3]. OLV provided a good surgical exposure of the collapsed lung while ensuring adequate gas exchange through the ventilated lung. Although DLT is the most used device to achieve these goals [13], BB was chosen because of the advantage of being placed through SLT and being more suitable for OLV in predicted DI [14].

The difficulty in the recognition of pain and the decreased expressive ability of SD patients make the use of self-report tools for pain assessment not advisable. Although it has exclusively been investigated in pediatric patients affected by DS, the COMFORT-B scale is the only one validated for this clinical population [15].

## Conclusions

To our knowledge, this is the first report describing the perioperative management of a DS patient who underwent thoracic surgery. The poor cooperation, difficult airways, and presence of comorbidities can challenge the anesthetic plan. An accurate preoperative assessment and adequate preplanned management, including premedication and advanced airway strategy, are required.
